# Looking Inside of the Intestinal Permeability Regulation by Protein‐Derivatives from Bovine Milk

**DOI:** 10.1002/mnfr.202400384

**Published:** 2024-11-12

**Authors:** Fabiola Guzmán‐Mejía, Daniel Efrain Molotla‐Torres, Marycarmen Godínez‐Victoria, Ximena Valdes‐Hilarios, Elizabeth Sánchez‐Miranda, Rigoberto Oros‐Pantoja, Maria Elisa Drago‐Serrano

**Affiliations:** ^1^ Departamento de Sistemas Biológicos Universidad Autónoma Metropolitana Unidad Xochimilco Calzada del Hueso No. 1100 Ciudad de México CP 04960 México; ^2^ Doctorado en Ciencias Biológicas y de la Salud Universidad Autónoma Metropolitana Calzada del Hueso No. 1100 Ciudad de México CP 04960 México; ^3^ Sección de Estudios de Posgrado e Investigación, Escuela Superior de Medicina, Instituto Politécnico Nacional Plan de San Luis y Díaz Mirón s/n Ciudad de México CP 11340 México; ^4^ Laboratorio de Neuroinmunoendocrinología Facultad de Medicina Universidad Autónoma del Estado de México Toluca 50180 Mexico

**Keywords:** bovine lactoferrin, colostrum, intestinal permeability, peptides, tight junction proteins

## Abstract

The prime function of the epithelium is to regulate the intestinal permeability; the latter is a quantitative parameter that refers to the measurement of the rate of passage of solutes through the epithelial monolayer. Function of epithelial monolayer depends on the expression of protein complexes known as tight junction proteins; whose function and expression can be disrupted under conditions of inflammation including irritable bowel disease (IBD), intestinal infections, and high‐fat diets, among others. This manuscript is focused to outline the effects of bovine milk protein derivatives on the intestinal permeability addressed mostly in animal models in which the intestinal barrier is disrupted. At present, the properties of bovine milk protein derivatives on intestinal permeability have been scarcely documented in humans, but evidence raised from clinical trials provides promising findings of potential application of colostrum to control of the intestinal permeability in critically ill patients, users of non‐steroid anti‐inflammatory drugs, like athletes and militia members.

## Graphical abstract

Intestinal barrier is determined by the intestinal epithelial monolayer, it acts as physical barrier, that allows the selective entry of nutrients and innocuous substances while hampers the access of potentially dangerous molecules. Function of epithelial monolayer depends on the expression of protein complexes known as tight junction proteins (TJPs) whose function and expression can be disrupted under conditions of inflammation as found in several chronic diseases like ulcerative colitis. Bovine milk derivatives and natural or synthetic lactoferrin peptides in intestinal permeability improve tight junction proteins localization and expression by molecular signaling mechanisms that involve the MAPK and NFκB pathways.

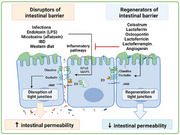

## 1 Introduction

Intestinal homeostasis denotes a balance resulting from the interplay of a wide array of events depending on microbiota, *mucus*, epithelium and subepithelial components of mucosal immunity; all these players support the functional and structural integrity of the gut barrier.^[^ ^1^ ^]^ Intestinal barrier function is determined by the epithelium that delimitates the lumen and subepithelial mucosal tissues allowing the selective entry of nutrients and innocuous substances while hampers the access of potentially dangerous molecules.^[^ ^2^ ^]^ The prime function of the epithelium is to regulate the intestinal permeability; the latter is a quantitative parameter that refers to the measurement of the rate of passage of solutes through the epithelial monolayer via paracellular and transcellular pathways.^[^ ^3^ ^]^ Intestinal epithelium is a single polarized cell monolayer that displays a luminal and basolateral membranes joined adjacently by transmembrane protexes including tight junction proteins (TJPs) expressed at most apical location of basolateral membrane of epithelial cells; TJPs determinate the paracellular permeability through both pore (size <8 Ǻ) and leaky (8–100 Ǻ) pathways.^[^ ^4^, ^5^ ^]^

Intestinal permeability is a dynamic process underwent to continuous changes of luminal components, including vitamins, probiotics, prebiotics and even, western style diet.^[^ ^3^ ^]^ Dairy products have driven the focus of attention as in the case of bovine colostrum a functional food that provides nutrients of high digestibility and absorptivity and also by containing components that exhibit modulatory properties in controlling intestinal permeability dysfunctions; therefore, colostrum has been proposed as supplement during the drug‐therapy in both clinical entities of the Intestinal Bowel Disease, i.e., ulcerative colitis and Crohn´s Disease, necrotizing enterocolitis and infant nutrition.^[^ ^6^, ^7^, ^8^, ^9^ ^]^ Colostrum is an enriched source of nutrients, cells, and potent bioactive molecules such as bovine lactoferrin (bLf).^[^ ^9^ ^]^ Bovine Lf displays prebiotics properties^[^ ^10^ ^]^ and whose multitask activities include its role in regulating intestinal barrier dysfunctions alleviating inflammation as seen in inflammatory bowel syndrome and even, cancer and neonatal intestinal maturation.^[^ ^11^, ^12^, ^13^, ^14^ ^]^

This manuscript was focused to outline the effects of bovine milk derivatives in intestinal permeability with special emphasis in quantitative assays of permeability. This approach may provide foundations on the design of pharmaceutical products containing bLf as ingredient to enhance the therapeutic efficiency and reduce the dosage of drugs prescribed for the treatment of disease of clinical relevance including irritable bowel disease (IBD). Furthermore, this contribution highlights the application of protein‐derivatives from bovine milk under conditions which gut permeability is disturbed, for example, in critically ill patients, users of non‐steroid anti‐inflammatory drugs and populations at high risk of physical and emotional stress like athletes, militia members, and active users.^[^ ^15^ ^]^

## 2 Intestinal Permeability

Intestinal permeability refers to the ability of the intestinal epithelium to regulate selectively the pass of substances toward the bloodstream; intestinal permeability is a quantitative parameter to measure the rate of passage of solutes through the epithelial monolayer via paracellular and transcellular pathways.^[^ ^3^ ^]^ Healthy intestinal permeability allows essential nutrients to be properly absorbed, while preventing toxins, bacteria, and other unwanted substances from entering the blood circulation. When intestinal permeability is altered, process known as “leaky gut,” unwanted substances can pass through epithelium and contribute to a variety of unhealthy conditions, including allergies, food intolerances, inflammatory, and autoimmune disorders as well as systemic disorders. There are generally two routes of intestinal permeability that are essential for maintaining intestinal homeostasis and nutrient absorption: the transcellular and paracellular pathways.^[^ ^3^ ^]^

Transcellular pathway involves transport of soluble molecules across epithelial cells takes place through simple diffusion and facilitated transport.^[^ ^16^, ^17^, ^18^ ^]^

Paracellular permeability is clinically relevant due to associated with various gastrointestinal conditions, such as IBD, celiac disease, and irritable bowel syndrome (IBS).^[^ ^19^ ^]^

Paracellular pathway involves the movement of substances between adjacent epithelial cells through the intercellular space where the TJPs complexes are located. Tight junctions regulate the passage of medium‐sized hydrophilic molecules (≤600 Da). However, paracellular route is impermeable to protein‐sized molecules avoiding the passages of antigenic macro‐molecules.^[^ ^18^ ^]^ Disruption of tight junction integrity can lead to increased paracellular permeability, allowing the passage of larger molecules, toxins, and pathogens from the intestine into the bloodstream condition known as “leaky gut.”^[^ ^18^ ^]^

### 2.1 Tight Junction Proteins

**Figure 1:**
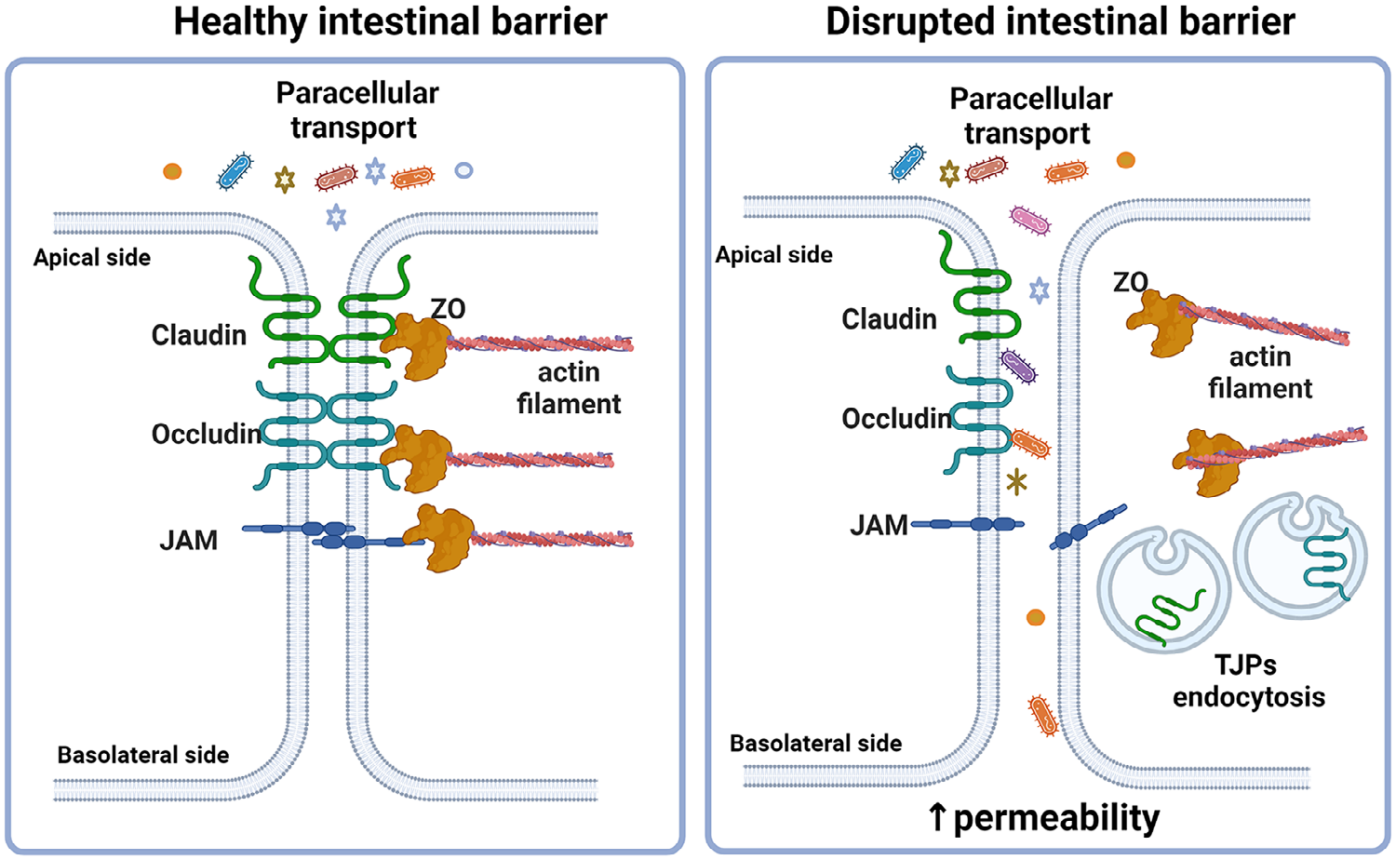
Location of TJPs in healthy and unhealthy conditions of the intestinal barrier.

The TJPs are protein complexes involved in maintaining the functional integrity of the intestinal barrier. Tight junctions consist of multiple proteins, expressed at the apical extreme of the basolateral membrane; TJPs interact each other to form a complex structure that links adjacent epithelial cells, regulating the passage of ions, molecules, and cells across epithelial barriers.^[^ ^20^ ^]^ Tight junction complex encompasses transmembrane proteins (occludin, claudins, and junctional adhesion molecules [JAMs]), cytoplasmatic scaffold proteins (zonula occludens [ZO], afadin, and cingulin) and cytoskeletal proteins (actin and myosin)^[^ ^4^, ^21^ ^]^ (**Figure** **1**).

Claudins (cldns) are a family of transmembrane proteins with at least 27 members in humans.^[^ ^18^ ^]^ Claudins are classified on base their sequence homology, tissue distribution, and functional properties. Functionally, cldns are divided into selective for cations such as cldn‐7, cldn‐10b, cldn‐2, and cldn‐15,^[^ ^22^ ^]^ or selective for anions for example cldn‐10 and cldn‐17.^[^ ^23^ ^]^ Also, there are some cldns with ambiguous channel properties, including cldn‐4, cldn‐7, and cldn‐16. Furthermore, cldns are classified as barrier‐forming cldns, which contribute to the decrease of the gut permeability, and as pore‐forming cldns, which favor the increase the gut permeability.^[^ ^22^ ^]^ Claudins have two extracellular loops (ECL 1 and ECL 2) that connect side by side the cells through several interactions including: cis, that refers the ELC union within the same cell, trans consists of the ELC union between adjacent cells, homophilic refers the ELC union of the same claudin family, while heterophilic implicates the ELC union among different claudin families.^[^ ^24^ ^]^

Occludin is a tight junction‐associated MARVEL tetraspan protein that exhibits a bicellular region that allows the trans‐homodimeric contact of the apical membranes. Both N‐ and C‐terminus of occludin are located at cytosol whereas C‐terminus exhibits phosphorylated residues for signaling kinases and a docking site for scaffolds. Occludin regulates the remodeling and location of TJP complex and contributes the TJ assembly in collaboration with scaffolding proteins such as ZO‐1. Occludin is involved in Tumor Necrosis Factor‐α (TNF‐α)‐induced Miossin Light Chain Kinase (MLCK)‐mediated increases in leak pathway permeability, thus occludin seems to contribute in the regulation of the paracellular permeability.^[^ ^4^, ^5^ ^]^

Zonula occludens (ZO) are cytoplasmic scaffold proteins that link the transmembrane proteins of tight junctions to the actin cytoskeleton inside the cell. Zonula occludens comprise ZO‐1, ZO‐2, and ZO‐3 that contain three tandems of PDZ structural domains. First PDZ1 domain interacts with cldn, second PDZ2 domain establishes dimerization with others PDZ2 domains, while PDZ3 regulates the bound by JAMs. Also, PDZ regions of ZO‐1 are coupled with other scaffolding proteins (cingulin and afadin), cytoskeletal components (actin and myosin), and occludin.^[^ ^4^, ^25^, ^26^ ^]^ Zonula occludens role is not determining for permeability regulation; however, it collaborates in mucosal repair and its down modulation in diseases like IBD disrupts mucosal healing.^[^ ^5^ ^]^ ZO proteins help anchor tight junction proteins to the cell membrane and regulate their assembly and disassembly. ZO proteins also play a role in signaling pathways that control tight junction function.^[^ ^27^ ^]^

Junctional adhesion molecules (JAMs) are transmembrane proteins of the immunoglobulin superfamily composed of an extracellular domain, a single‐pass transmembrane region, and a short cytoplasmic tail.^[^ ^28^ ^]^ There are four types of JAM, namely, JAM‐A, JAM‐B, JAM‐C, and JAM‐4. Homodimers of JAM‐A connect opposing cell surfaces ensure intercellular interaction through the N‐terminal immunoglobulin‐like domain, while their C‐terminus PDZ‐motif binding interact with cytoplasmic scaffold proteins (ZO and afadin) and cytoskeletal proteins. JAMs proteins have a role in stabilizing tight junctions and regulating the paracellular permeability for macromolecules.^[^ ^4^, ^29^ ^]^

Accordingly, the TJPs coordinate the interaction between transmembrane, scaffold, and the cytoskeleton proteins (actin, afadin, microtubules, and myosin), as well as mediate signaling pathways that control tight junction function.^[^ ^27^, ^29^, ^30^ ^]^ Under some conditions, TJPs can be endocytosed through mechanisms that involve the activation of various cytoskeletal proteins, increasing the permeability of the intestinal barrier (Figure 1).^[^ ^31^, ^32^ ^]^

### 2.2 Methods for Intestinal Permeability Assessment

As mentioned before, intestinal permeability takes places through two pathways, the transcellular and paracellular pathways. In addition, at research and clinical levels. Also, there are several methods to evaluate both permeability routes, the paracellular and transcellular pathways.^[^ ^18^ ^]^ In **Table** **1** are mentioned some methods used in the clinical practice to evaluate the paracellular permeability.

**Table 1:** Methods to measure the paracellular permeability from intestinal lumen to bloodstream in clinical practice and research.

Methods used for clinical research		
Method	Procedure	Comments
Using chamber technique (UCT)	Ex vivo technique that involves a device which the human or animal intestinal tissue is mounted between two chambers containing ions, solutes, or macromolecules to measure their movement across tissue over time.	UCT is useful to assess the selective paracellular permeability of ions by TEER (Ω cm^−2^). UCT allows to monitor electrophysiological parameters (potential difference, short circuit current, and TEER).^[^ ^18^, ^33^ ^]^
Epithelial Voltohmmeter device (EVOM)	In vitro technique in culture of several intestinal cell lines to measure high transepithelial electrical resistance (TEER).	This technique is used to evaluate the paracellular permeability.^[^ ^34^ ^]^
FITC‐dextran conjugate (3–2000 kDa)	In vivo technique to measure of fluorescence in plasma samples after oral administration of dextran by fluorescence spectroscopy.	Permeability to high size of FITC‐dextran reflects damage to TJP structure.^[^ ^35^, ^36^ ^]^

Methods for clinical use		
Sugar absorption tests [lactulose and mannitol or lactulose‐ rhamnose].^[^ ^37^, ^38^ ^]^	Measurement of amount of sugar absorbed from the intestine into the bloodstream at specific intervals, after oral administration.	These procedures allow to evaluate the paracellular transport in intestinal diseases and stress‐response associated to exercise.
Lactulose and mannitol excretion test in urine.^[^ ^39^ ^]^	Measurement of amount of sugar excreted in urine over a specified period, after oral administration.	
Permeability probe test [polyethylene] glycol (PEG) of different molecular sizes.^[^ ^37^ ^]^	Determination of amount of PEG excreted in urine after oral administration.	
^51^Cr‐ethylenediaminetetra‐acetic acid (EDTA)/^14^C‐mannitol methods.^[^ ^39^, ^40^ ^]^	Measurement of marker concentration excreted in urine after oral administration.	
Zonulin^[^ ^41^ ^]^ lipopolysaccharide‐binding protein.^[^ ^42^ ^]^	Enzyme‐linked immunosorbent assay (ELISA) technique Measurement of these markers in blood samples can provide indirect evidence of altered intestinal permeability and gut barrier dysfunction.	Measurement of these markers in blood samples can provide indirect evidence of altered intestinal permeability and gut barrier dysfunction. Although, in health conditions is not reliable marker.^[^ ^43^ ^]^
Endoscopic technique.^[^ ^44^ ^]^	Direct visualization and assessment of intestinal barrier function at the cellular level in vivo. This technique provides real‐time information about epithelial integrity and permeability during endoscopic procedures.	Confocal laser endomicroscopy and fluorescein‐labeled dextran imaging.
Electrical Impedance Spectroscopy (EIS) combined with electrical stimulation.^[^ ^45^ ^]^	Noninvasive technique that measures the electrical impedance of tissues to assess their integrity and permeability.	Applied to the gut and intestine, EIS can provide real‐time information about transepithelial barrier function and tissue structure of human intestinal epithelium cultured inside an organ‐on‐chip microfluidic culture device.

Accordingly, the assessment of the intestinal paracellular permeability from intestinal lumen to bloodstream is crucial for the evaluation of function of gut barrier on pathologic conditions, with aim of improve the clinic treatment.

## 3 Bovine Milk: Proteins and Peptides

Bovine milk is an exocrine secretion from mammary gland cells regarded as a functional food by providing nutrients and by containing bioactive components that are beneficial for intestinal health.^[^ ^46^, ^47^ ^]^

Milk is enriched source of nutrients including lactose, lipids, minerals and vitamins, proteins, and water. The major sugar in milk is lactose, which provides the energy that is vital for surviving of the neonate mammal. In milk, the lipids are mostly in the form of fat globules surrounded by a membrane of polar lipids and proteins namely as milk fat globule membrane (MFGM) that contain triacylglycerides as main molecules located within the core of MFGM. Other milk fats include free fatty acids, cholesterol, mono and diacylglycerides, phospholipids, and fat soluble vitamins like vitamin A and E that acts as anti‐oxidants.^[^ ^46^, ^47^ ^]^ Furthermore, prominent minerals present in milk include calcium and phosphorous with critical role in bone structure, magnesium involved in muscle contraction and selenium important in the immune system.^[^ ^46^, ^47^ ^]^

In regard other nutrients, milk is a high‐quality source of dietary proteins for humans with a wide array of biological actions such as antimicrobial, growth factors, hormones, antibodies, and immune regulators. Two major classes of protein are present in milk: casein (80 %) and whey proteins (20%). Whey fractions include β‐lactoglobulin, α‐lactalbumin, bLf, bovine serum albumin, and immunoglobulins. Milk also contains a complex enzyme mixture of proteases, zymogens, protease activators, and protease inhibitors.^[^ ^48^ ^]^

Milk proteins are digested at the stomach by pepsin and cathepsin D which are activated by the high acidity provided by the hydrochloric acid. This enzymatic protein degradation is the first key step, which is followed by further digestion in the luminal small intestine with the addition of pancreatic proteases like trypsin and chymotrypsin, to be terminally degraded by brush border exopeptidases. In the colon, microbes can contribute to further proteolysis of undigested milk proteins.^[^ ^48^ ^]^

Milk peptides derived from the intestinal digestion have biological activity, such as lactoferricin, released from the full length bLf proteolysis.^[^ ^49^ ^]^ It is worth to indicate that, Lf and osteopontin are slightly degraded in intestinal tract what allows and optimal bioavailability therefore favoring their biological action.^[^ ^50^ ^]^

The next section describes some characteristics of bioactive components of milk with modulatory actions in intestinal permeability.

### 3.1 Colostrum

Bioactive components of milk, such as α‐lactalbumin, immunoglobulins, and bLf are present in higher concentrations in the colostrum than in the mature bovine milk, suggesting a significant contribution to neonatal nutrition.^[^ ^51^ ^]^

Colostrum and mature milk have very similar framework nutrients, but colostrum contains a greater amount of immunoglobulins (IgA, IgM, IgE, IgD, IgG), proteins (casein and Lf), milk‐protein‐derived peptides (glycomacropeptide, casecidin, isracidin, peptides), cytokines such as TNFα, interleukin 1β (IL‐1β), and interferon γ (IFN‐γ), growth factors including Transforming Growth Factor‐β (TGF‐β), Platelet Derived Growth Factor (PDGF), Vascular Endothelial Growth Factor (VEGF), and Fibroblast Growth Factor 2 (FGF2), and enzymes (xanthine oxidase, lactoperoxidase, and ribonuclease.^[^ ^51^ ^]^ The availability of these factors has demonstrated reduction of intestinal permeability as shown in murine models of inflammatory diseases; however, the underlying mechanisms on the anti‐inflammatory effects have not been thoroughly explained.^[^ ^2^, ^52^ ^]^

### 3.2 Lactoferrin

Lactoferrin (Lf) is an iron‐binding multifuncional glycoprotein of 80 kDa that belongs to the transferrin family; present as a cationic protein at physiological pH 7, with an isoelectric point of 8.0–8.5.^[^ ^53^, ^54^ ^]^ In milk, Lf is present as an iron‐free form known as apo‐Lf and as iron‐loaded form namely as holo‐Lf.^[^ ^2^ ^]^ Lactoferrin has a higher purity when is isolated from the mature milk than the colostrum, moreover, its concentration is higher at the colostrum; differences in purity and concentration can affect the biological effects.^[^ ^55^ ^]^ Anti‐inflammatory and antimicrobial actions of Lf have been ascribed to the ability of binding to microbial molecules, for example, lipopolysaccharide (LPS), lipoteichoic acid, heparan sulfate, DNA, and RNA.^[^ ^54^, ^56^ ^]^ Moreover, Lf plays an important role in the intestinal health, not only in neonatal development by promoting maturation of the neonatal small intestine and increasing the integrity of the gut barrier,^[^ ^14^ ^]^ but also in adult wellbeing due to its anti‐inflammatory effects on multiple intestinal pathologic processes.^[^ ^52^, ^57^, ^58^ ^]^

### 3.3 Lactoferricin

The enzymatic hydrolysis of human or Lf renders some peptides with greater antimicrobial activity than Lf itself, such as Lactoferricin (Lfcin), a cationic peptide with molecular weight around 39 kDa.^[^ ^49^ ^]^ Lactoferricin consists of an amino acid loop formed by a disulfide bridge between cysteine residues.^[^ ^59^ ^]^ Bovine lactoferricin retains the N‐terminal region of bLf, which is involved in immunomodulatory functions through binding to surface components of mammalian cells and antimicrobial properties by binding to the microbial compounds, but lacks iron binding property.^[^ ^49^ ^]^ Currently, various analogues of Lfcin had been identified and are classified according to their structure, which is determined by the number of amino acids derived from the hydrolysis of bLf by proteases such as pepsin^[^ ^60^ ^]^ and chymosin;^[^ ^61^ ^]^ both enzymatic proteolysis produce peptide derivatives from the N‐terminal region of parental Lf.^[^ ^49^ ^]^ Lactoferricin has a wide antimicrobial spectrum which includes Gram‐negative and Gram‐positive bacteria, yeasts, filamentous fungi, and parasitic protozoa.^[^ ^49^ ^]^ In addition, Lfcin can attenuate damage to the epithelial barrier by reducing inflammation and improving the expression of ZO‐1 and occludin as seen in a model of intestinal damage with zoonotic enterohemorrhagic *Escherichia coli* (EHEC).^[^ ^62^ ^]^ Lactoferrampin (LFA) is peptide identified in the N‐terminal lobe of bLf that contains 17‐residue corresponds to 268–284 of bLf. Lactoferrampin is not a natural peptide that displays antibacterial and candidacidal activity. Moreover, LFA prevents the intestinal damage as documented in Dextran Sodium Sufate (DSS)‐induced murine colitis model.^[^ ^63^ ^]^

### 3.4 Osteopontin

Aside of Lf, additional milk bioactive proteins such as osteopontin also display modulatory properties in the intestine. Osteopontin is a matricellular protein of 66 kDa present in higher concentration in colostrum than in mature milk.^[^ ^64^, ^65^ ^]^ Osteopontin is prone to generate complexes with other lactoproteins such as Lf.^[^ ^65^ ^]^ Unlike the separated proteins the Lf‐osteopontin complex has greater resistance to enzymatic hydrolysis and it is readily absorbed in intestinal cells.^[^ ^65^ ^]^ Moreover, Lf‐osteopontin complex has synergistic actions in the repair of damage to the intestinal barrier induced with LPS.^[^ ^50^ ^]^ Osteopontin has shown to give strength to the intestinal barrier by improving the permeability and by reducing the inflammatory response as found in DSS‐induced colitis mice.^[^ ^64^, ^66^ ^]^

Along with Lf and osteopontin, the angiogenin is another immunomodulatory protein present in milk of 14 kDa. Angiogenin has a wide range of biological effects, such as RNase activity, induction of blood vessel growth, regulator of healing, embryogenesis among others.^[^ ^67^ ^]^ Currently, the only one study has demonstrated that angiogenin improves the intestinal permeability by reducing the intestinal inflammation induced by LPS.^[^ ^68^ ^]^ Future studies should be done to address to role on the angiogenin in the intestinal permeability.

### 3.5 Milk Derivate Peptides and Microbiota

Milk is a source of both probiotics referred as live microorganisms, and prebiotics that are compounds which consume enrich the diversity and abundance of the intestinal microbiota.^[^ ^69^ ^]^ Microbiota orchestrates the microbial antagonism that hampers the overgrowing and colonization of pathogenic microorganisms and importantly, microbiota release metabolites that regulate the intestinal barrier.^[^ ^70^ ^]^ Microbiota population growing can be influenced by milk protein and peptides “embellished” with β‐N‐glycans regarded as prebiotics that act as energy sources that increase the growing of selected microbiota populations.^[^ ^71^ ^]^ Bovine milk oligosaccharides decrease the intestinal permeability and ameliorate dysbiosis as documented in diet‐induced obese mice.^[^ ^72^ ^]^ Milk derivate peptides, such as Lfcin, provide benefits by re‐enforcing the structural integrity of TJPs and by decreasing the abundance of potentially pathogenic microbiota members (*Bacteroides*, *Barnesiella*, and *Escherichia*).^[^ ^73^ ^]^ In addition, Lf enhances the abundance of *Clostridium* and triggers the microbiota metabolite concentration, such as butyrate, associated with the improvement of intestinal permeability.^[^ ^74^ ^]^ Interplay between Lf and microbiota upon the modulation of the intestinal permeability was evidence in Lf knock‐out mice (Wang, 2023) and zonulin transgenic mice.^[^ ^75^ ^]^

Accordingly, proteins present in the milk provide a wide range of beneficial effects on reinforcement on the regulations of the intestinal barrier components as describe in the next sections.

## 4 Bovine Milk Derivatives and Intestinal Permeability

Given their pharmaceutical and clinical impact, in vitro studies have been focused to analyze the regulatory properties of bLf and dairy products like colostrum and whole milk on gut barrier and other aspects like intestinal maturation, neurodevelopment among others. Under in vitro conditions, critical issues entail the analysis of bLf properties on gut permeability including the type of cell monolayer used for culture assays, extent of bLf iron‐saturation and source for bLf purification or contained in colostrum or milk collection whether early‐ or mid‐term lactation.

Findings from in vitro cultures of Caco‐2 and human epithelial crypt cells (HIECs) cell lines evidenced that in both cell lines, bLf promoted both the alkaline phosphatase activity as marker of intestinal maturation and the cell growth by arresting the cell cycle at the G2/M‐phase. Bovine Lf improved the physical epithelial barrier evidenced by decreasing the transport of sodium fluorescein in a dose‐dependent manner and by upmodulating the transepithelial electrical resistance (TEER), the TJP mRNA levels (*CLDN1*, *OCLDN*, *TJP1*), as well as TJP expression (cldn‐1, occludin, and ZO‐1). In comparison with Caco‐2 cells, HIEC monolayer was less sensitivity to the upmodulating effect of bLf on cell growing and showed TEER values similar to that found in human epithelial cells; findings indicated that HIEC cells resembled closely the human epithelial monolayer therefore can be a better model than Caco‐2 to assess under in vitro condition the action of bLf in gut barrier components in human epithelial cells.^[^ ^76^ ^]^ Additional studies in established Caco‐2 cell monolayers using TEER permeability assay showed that treatment with, bovine, native and recombinant human Lf (100 µg mL^−1^ 24 h^−1^), had no significant effect on the Caco‐2 cell monolayer barrier model. However, these treatments promoted an increase in TEER value during the process of establishment of Caco‐2 cell monolayers.^[^ ^77^ ^]^

In other studies, in Caco‐2 culture assays, the TJPs reinforcement associated to TEER increase was found only when bLf is present in whole colostrum but not in purified form; unlike bLf contained in whole colostrum, purified bLf did not mitigate the loss of barrier function evidenced by reduced TEER values in Caco‐2 cells challenged with TNF‐α as inductor of gut barrier damage.^[^ ^78^ ^]^ Finding may suggest that effect of bLf on gut barrier may be potentiated by additional components in milk like angiogenin and osteopontin as outlined below.^[^ ^50^, ^55^ ^]^

Findings from in vitro cell cultures indicated that unlike the one isolated from mid‐lactation milk the bLf isolated from early‐lactation milk, was capable of increasing TEER in Caco‐2 monolayers and this effect was ascribed to its ability of reducing the amount of interleukin 8 (IL‐8) tested in peripheral blood mononuclear cell culture.^[^ ^55^ ^]^ Apparently, bLf properties on the reinforcement of gut permeability was associated to the contamination of angiogenin eluted during the bLf purification from early‐lactation milk samples. Angiogenin is a peptide with antimicrobial activity and induces the expression of cldn‐1, occludin‐1, and ZO‐1 so that improves the intestinal barrier function mainly at jejunum.^[^ ^68^ ^]^ Thus, angionenin present in milk may be beneficial maintaining the gut barrier integrity.

Although, the activity of bLf might be dependent on other milk components, animal models of experimentation provided additional insights about the impact of bLf on gut barrier regulation. The role of bLf during perinatal and neonatal development has been documented in neonatal rat pups delivered from rat moms receiving standard diet containing bLf during mating, gestation and lactation.^[^ ^79^ ^]^ This assay showed that oral ingested bLf only altered some intestinal permeability parameters (TEER and transepithelial conductance) in colon not in ileum without changing others (permeability to fluorescein isothiocyanate [FITC]‐dextran 4000 [FD4]) short‐circuit current, ΔIsc glucose in both intestinal segments from rat pups. Bovine Lf supplementation increased the gene expression of occludin, cldn‐2,‐5, and ‐7 in colon, and only occludin and cldn‐15 in ileum. Furthermore, Lf supplementation increased both the body and brain weight in rat pups without affecting the protein brain derived neurotrophic factor (BDNF) as marker of brain maturity. Findings indicated that bLf strengths gut barrier integrity mainly in colon and provides beneficial effects on neonatal intestinal and neurodevelopment.^[^ ^79^ ^]^

Additional aspects of Lf on gut barrier have been documented in breast‐fed neonatal mice delivered by Lf (Lft)‐knockout female mice and then underwent chronic unpredictable mild stress. Results evidenced that in the adult life, Lf‐feeding deficiency increased the LPS translocation from the intestinal lumen to systemic circulation and the serum levels of TNF‐α and IL‐1β, decreased the levels of BDNF; the mechanism accounted the outcome of Lf depletion was ascribed to overactivation of inflammatory response elicited by the interaction of LPS and toll‐like receptor 4 (TLR4) at intestinal and brain levels.^[^ ^14^ ^]^ Data highlight the role on “microbial‐intestine‐brain” axis in the regulation of intestinal permeability for intestinal wellness.

Dairy products such as colostrum, milk, and bLf exhibit modulatory properties on intestinal permeability contributing to the protection intestinal maturation, neuronal development, enteric infections in infants, and chronic inflammatory diseases like ulcerative colitis.^[^ ^6^, ^7^, ^80^ ^]^

### 4.1 Effects of Lactoferrin and Milk Derivatives on Intestinal Permeability Caused by Microorganisms

It has been shown that bLf, Lfcin, osteopontin, and some dairy derivatives can have direct effects on improving intestinal permeability and counteract the deleterious impact on intestinal permeability of some stimuli including LPS, micotoxins, and bacterial infections as described following.

In vitro settings have analyzed the extent of iron saturation of bLf on gut barrier of Caco‐2 and J774A.1 macrophage cell line cultures treated with holo‐(iron saturated) or apo‐(iron‐depleted) bLf.^[^ ^81^ ^]^ Both Lf forms did not show apoptotic activity nor changed the protein expression of TJP in Caco‐2 cells. Both Lf forms, mainly apo‐Lf, ameliorated the increase of inflammatory cytokines such as IL‐6 and TNF‐α in LPS‐primed macrophage cell cultures. Anti‐inflammatory outcome was attributed to the ability of bLf to bind and to neutralize LPS;^[^ ^82^ ^]^ it is known, cognate ligation of LPS with TLR4 elicits nuclear factor κB (NFκB) signal pathway resulting in the generation of inflammatory cytokine responses.^[^ ^83^ ^]^ In addition, bLf may enter via LRP to cytosol and bind to TNF receptor associated factor (TRAF6) and prevent the activation of proteins involved in mitogen activated protein kinase (MAPK) and IkappaB kinase (IKK‐IκBsignaling pathways.^[^ ^84^ ^]^ Inflammatory cytokine like TNF‐α causes weakening of the gut barrier through signaling pathways involving the activation of MLCK leading caveolin‐1‐dependent occludin internalization and contraction of cytoskeleton.^[^ ^4^ ^]^ Even though in this study holo‐Lf and apo‐Lf did not enhanced the TJP expression, data indicated that both preserved the TJPs integrity and the ability of apo‐Lf to neutralize LPS driving the reinforcement of the intestinal barrier function in high risk populations like neonates and infants.^[^ ^81^ ^]^

Other studies based on in vitro assays in human intestinal Caco‐2 cells showed that treatment with bLf at doses of 400 and 1000 mg mL^−1^ inhibited the enhancement of LPS‐mediated permeability assessed by measuring TEER and FITC‐labeled dextran 4000 (FD‐4) after 2 h of LPS treatment.^[^ ^85^ ^]^ Also, in other study in Caco‐2 cell monolayers TEER and FITC‐dextran permeability were measured in presence of LPS. After 24 h of treatment, LPS at 1, 10, and 100 µg mL^−1^ significantly reduced the TEER value in a dose‐dependent manner and increased the FITC‐dextran permeability.^[^ ^77^ ^]^ The dose 100 µg mL^−1^ of LPS was used to establish the LPS‐damage barrier model and to evaluate the effect of Lf treatment. The preincubation of cells with human Lf, bLf, and recombinant human Lf for 24 h reduced the decrease in TEER and the increase of FITC‐dextran permeability induced by LPS.^[^ ^77^ ^]^

Osteopontin is a glycosylated protein secreted in milk whose multifunctional properties including regulation of the junctional distribution of occludin resulting in the maintenance of TJPs integrity.^[^ ^50^, ^66^, ^86^ ^]^ In vivo model of LPS‐induced intestinal barrier injury in C57BL/6 mice showed that oral administration of bLf‐osteopontin combination induced protective effect on the intestinal barrier promoting a decrease of intestinal permeability markers such as serum diamine oxidase (DAO) activity and D‐lactic content, resulting in the rate reduction of FITC‐labeled glucan transport across the jejunum; furthermore, the bLf‐osteopontin combination improved ZO‐1, occludin, and cldn‐1 protein expression and mRNA levels in the intestinal epithelium.^[^ ^50^ ^]^

Studies based on in vitro assays in Caco‐2/HT‐29 co‐cultures treated with cytomix (cocktail of TNF‐α, IL‐1β, and IFN‐γ) as inductor of inflammatory injury, analyzed the impact of two milk‐isolated derivatives bLf and osteopontin and whey protein concentrate on gut barrier integrity.^[^ ^86^ ^]^ In this assay cytomix‐induced TEER decrease was counteracted by bLf and whey protein concentrate but in greater extent by osteopontin. In regard TJPs analysis, down‐modulating effect of cytomix on ZO‐1 and occludin mRNA levels was mitigated by all samples although cldn‐3 decrease was only counteracted by osteopontin. At protein level, cytomix‐induced downmodulation of ZO‐1, occludin, and cldn‐3 was only counteracted by osteopontin not by bLf neither whey protein concentrate. Analysis of inflammatory cytokines indicated that citomix‐induced IL‐8/IL‐6 mRNA/protein up‐modulation was counteracted by bLf and whey protein concentrate but in greater extent by osteopontin. Anti‐inflammatory outcome was associated with decreased mRNA levels of NFκB pathway activation (MyD88, NFκB p65, IκB‐α).^[^ ^86^ ^]^ Thus, findings indicated supplementation of diet containing bLf and osteopontin may reinforce the gut barrier with implications in protecting enteric infectious and chronic inflammatory intestinal diseases.

In other in vitro assays, using infection or inflammation models, the actions of Lf on intestinal permeability had been described. It is know that TNFα decreases TEER and increases the permeability of fluorescein and FITC–dextran (4 kDa) as found in the human epithelial HT‐29/B6 and T84 cell cultures.^[^ ^87^ ^]^ The preincubation for 3 h with 0.5 mg mL^−1^ bLf attenuated the TNF‐α‐induced TEER decrease after 24 h, also bLf restored tight junction morphometry evaluated by freeze‐fracture electron microscopy; moreover, bLf incubation did not prevent TNF‐α‐induced redistribution of cldn‐1 and cldn‐2 nor enhanced the localization of cldn‐3 or cldn‐4 evaluated by confocal microscopy. In the same study, in cell cultures infected with the enteropathogenic bacterium *Yersinia enterocolitica* decreased TEER was seen in HT‐29/B6 cell monolayer within 48 h after infection and in T84 monolayers within 9 h after infection. The effects of bLf at 0.2 and 0.5 mg mL^−1^ doses in both cell epithelial monolayers ameliorated the *Yersinia enterocolitica*‐induced TEER decrease. However, bLf was not effective in revert the *Campylobacter jejuni*‐induced TEER drop tested only in T84 monolayers. Also, bLf prevents the *Y. enterocolitica*‐induced reduction of cldn‐8 protein levels and the underlying mechanism resulted from the molecular signaling pathways associated with the decrease of phosphorylation status of c‐Jun kinase generated by bLf.^[^ ^87^ ^]^

The protective effects of bLf on intestinal permeability had been observed in derivatives products such as the Pepsin‐treated Lf (PLF) and Lfcin B. In an in vitro study in rat intestinal epithelial (IEC‐6) cells stimulated with toxin B produced by *Clostridium difficile* was observed that toxin B inhibit cell wound restitution, ZO‐1 redistribution, and decreased of expression of ZO‐1 and occludin. The addition of Lfcin B and PLF to toxin B‐treated cells significantly increased wound cell restitution and counteracted the toxin B induced decreased expression of ZO‐1 and occludin.^[^ ^88^ ^]^

In addition to having effects on bacterial toxins, some dairy derivatives also have effects on alterations in permeability caused by mycotoxins. In in vivo model using deoxynivalenol (DON) mycotoxin for causing intestinal dysfunction shows that DON increases the jejunal expression of occludin, proinflammatory cytokine IL‐1β, and villus height. The results of this study indicated that the oral treatment with bLf (10 mg 100 µL^−1^) prevented the intestinal damages DON‐induced.^[^ ^74^ ^]^ Also, actions of Lf on cells exposed to aflatoxin, mycotoxins that are produced by several strains from the *Aspergillus* family, have been explored. In vitro assay conducted in differentiated Caco‐2 cells exposed to aflatoxin‐1 (AFM1) showed that bLf treatment decreased the AFM1‐induced increase intestinal permeability assayed as TEER and bLf improved the gene expression of cldn‐3, occludin and ZO‐1, and repaired the AFM‐1‐injured intestinal barrier. Results of the of the paracellular flux with lucifer yellow (LY), showed that bLf suppressed the paracellular passage of LY, improving the intestinal barrier function.^[^ ^89^ ^]^ The underlying pathways that explained the results on permeability were explored using bioinformatics resources, and reported that both individual and combined AFM1 and bLf treatment regulated the intestinal cell‐related signaling pathways, such as apoptosis, cell cycle, MAPK, and Wnt signaling pathway, regulation of actin cytoskeleton, focal adhesion, adherents, tight and gap junctions.^[^ ^89^ ^]^

Furthermore, Lfcin effects in intestinal permeability had been evaluated in the murine model of *E. coli* (EHEC) O157:H7‐induced intestinal dysfunction. In this infection model the mucosal architecture of jejunum was characterized by fewer intestinal villi and damage in the intestinal epithelium; also, *E. coli* O157:H7 infection induced the decrease of ZO‐1, cldn‐1, occludin, Muc‐1, and Muc‐2 mRNA levels and protein expression, as well as, TEER evaluated in the Ussing chamber. The Lfcin B treatment by gavage (0.5 mg kg^−1^ body) improved the protein expression of TJPs and prevented the reduction of TEER induced by *E. coli* O157:H7 infection.^[^ ^62^ ^]^

According to the evidence, bLf and derivatives showed beneficial effects on intestinal permeability against the damage induced by infections and toxins, but future studies may provide additional evidence about the specific of molecular signaling pathways.

### 4.2 Effects of Lactoferrin and Milk Derivatives on Intestinal Permeability Caused by Inflammatory Bowel Disease

Irritable bowel disease (IBD) is a common, long‐term condition of the digestive system, characterized by chronic inflammation of the digestive tract and includes ulcerative colitis and Crohn's disease.^[^ ^90^ ^]^ Ulcerative colitis involves the inflammation and ulceration of the superficial layers of the large intestine (colon) and rectum usually accompanied by disruption of the intestinal mucosal barrier and increment of permeability, causing invasion by microorganisms that exacerbate colon inflammation.^[^ ^91^ ^]^ There is some evidence that bLf derivatives counteract the harmful effects on intestinal permeability caused by alterations of the intestinal barrier in chronic inflammatory diseases.^[^ ^52^, ^63^, ^73^, ^92^ ^]^ Many animal models of ulcerative colitis have been generated to study the physiopathology of disease, including the DSS model.^[^ ^93^ ^]^

Murine model of ulcerative colitis induced by DSS provides in drinking bottles evidenced that mice in the DSS treated group exhibited severe intestinal epithelial tissue damage with a large infiltration of inflammatory cells and decrease of ZO‐1, cldn‐1, and occludin expression in colon tissues. The results showed that ZO‐1 expression in mouse colon tissues, examined by immunofluorescence staining, was significantly reduced in the DSS‐group, and was prevented by Lfcin B the treatment for 3 days (oral administration 5 mg kg^−1^). The expression of the three TJPs, analyzed by Western blotting ZO‐1, occludin, and cldn‐1 in the DSS‐group was decreased, but the Lfcin treatment reversed the DSS‐induced decreased only of ZO‐1 and cldn‐1 expression. To evaluate the molecular signaling pathways involved in the effects of Lfcin on inflammation caused by DSS, this study analyzed the protein expression of molecules belonging to NFκB and MAPK pathways. The results indicated that, the relative expressions of p‐p65, p‐ERK, p‐JNK, and p‐p38 were all highly significantly up‐regulated in mice after DSS ingestion, and after Lfcin B treatment, the relative expression of p‐p65 and p‐p38 was highly significantly down‐regulated, nevertheless, Lfcin treatment did not change the expression of p‐ERK and p‐JNK.^[^ ^73^ ^]^ In another study, the effects of bovine lactoferricin–lactoferrampin encoding *Lactococcus lactis* strain (LL‐LFCA) administrated intragastrically on days 1–7 at a concentration of 5 × 10^9^ colonic forming units (CFU) could decrease the DSS‐induced intestinal permeability increase evaluated by DAO and LPS concentration in plasma samples.^[^ ^63^ ^]^ The expression levels of the TJPs ZO‐1, E‐cadherin, and cldn‐2 in the colon, assessed by RT‐PCR and western blotting, indicated that the administration of LL‐LFCA in DSS‐treated mice enhanced the mRNA expression levels of E‐cadherin and cldn‐2, but not ZO‐1. The LL‐LFCA treatment ameliorated DSS‐induced colon damage, moreover, inhibited the inflammatory cell infiltration and decreased myeloperoxidase activity.^[^ ^63^ ^]^

Finally, it is important to mention that there are other conditions less explored that can alter intestinal permeability, for example, short bowel syndrome and a high‐fat diet. In both, the bLf treatment has been shown to improve intestinal barrier function.^[^ ^57^, ^58^ ^]^ In a model of short bowel syndrome (SBS), in rats submitted to 80% small‐bowel resection, the treatment with bLf (oral administration, 0.5 g kg^−1^ of body weight from day 2 to day 20) decreased the bacterial translocation to regional organs and intestinal permeability, evaluated by orally administration of permeability markers containing lactulose/mannitol (L:M) for evaluation in urine samples, was significantly reduced. Treatment of bLf also improved the expression level of occludin and cldn‐4 compared with SBS group. These reports evidenced the beneficial role of bLf on the treatment of SBS.^[^ ^58^ ^]^ In regard the effects of diet models that alter the intestinal permeability, the high‐energy‐dense Western‐style diets rich in fat and simple carbohydrates contribute to the development of obesity associated diseases that alter the intestinal barrier, partly due to the decrease in the expression of TJPs. A study using a rat model of obesity generated by the consumption of a Western diet demonstrated that bLf treatment, intragastric administered 500 mg kg^−1^ body weight five times a week, upregulated the protein expression of ZO‐1 and occludin, and increased the abundance of *Bacteroidetes* at phylum and *Roseburia* at genus in colonic tissue. This evidence suggested that the bLf can be regulated the permeability altered by high fat diets.^[^ ^57^ ^]^

**Figure 2:**
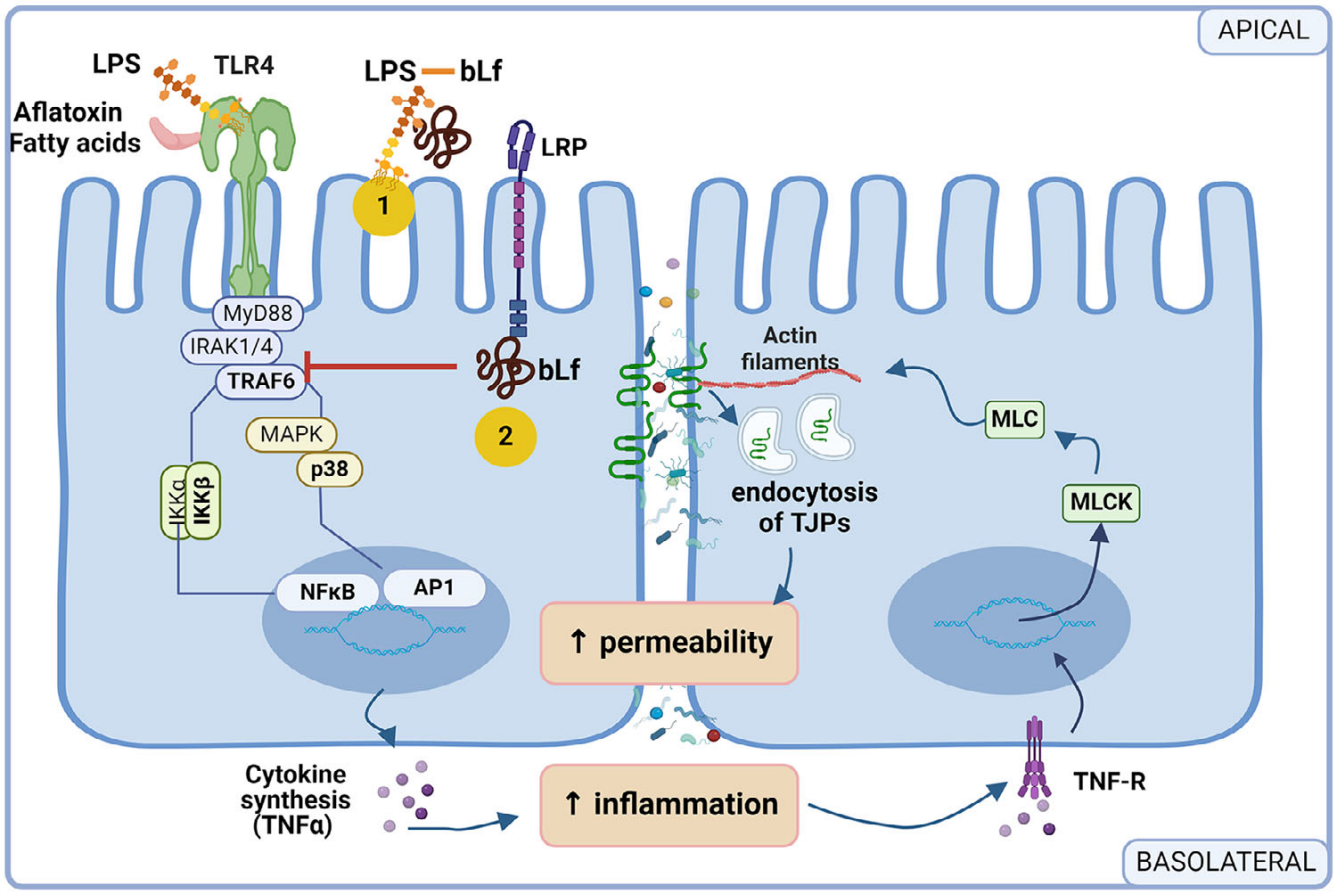
Possible molecular mechanisms of bLf regulation on intestinal permeability alterations induced by the activation of the TLR4. (1) Capture of LPS by bLf and (2) inhibition of TRAF6 by intracellular bLf.

The evidence described previously indicates that the activation of the IKK‐NFκB and MAPK signaling pathways mediate the damage caused to intestinal permeability by deregulating the localization and expression of TJPs. It is possible that under conditions of bacterial or fungal infection or a high‐fat diet, the activation of the TLR4 receptor by ligands such as LPS, aflatoxin or fatty acids induces the activation of these signaling pathways, promoting inflammatory cytokines production, such as, TNF‐α and INF‐γ that alter TJPs expression and location.^[^ ^32^, ^94^ ^]^ There are studies that indicate that TNF‐α, by activating its receptor in epithelial cells, induces the expression of MLCK, which phosphorylates the MLC protein to promote the displacement of actin filaments altering the localization of TJPs^[^ ^32^ ^]^ and causing their endocytosis increasing of intestinal permeability (**Figure** **2**).

Colostrum and other immunomodulatory proteins from milk have beneficial effects on the intestinal barrier; however, the molecular mechanisms associated with this regulation have been little explored. Two approaches have been described, one of them implicates the bLf binding to LPS,^[^ ^82^ ^]^ preventing the interaction of endotoxin to TLR4. The second mechanism involves the transport of Lf via LPR that allows the intracellular location of bLf blocking TRAF6.^[^ ^84^ ^]^ In both pathways Lf prevent intracellular TLR signaling pathways (Figure 2).

## 5 Effects of Colostrum in Human Intestinal Permeability

Growing number of in clinical trials studies have evidenced the immunomodulatory and antibacterial properties of milk derivatives proteins such as Lf. In the clinical field the intestinal permeability effects of Lf, Lfcin, osteopontin, angiogenin, and other derivative proteins of milk have not been explored. Thus, at present, only the properties of colostrum on intestinal permeability in humans have been scarcely documented as described in the next section.

Modulatory effects of colostrum and bLf on gut permeability have been analyzed in humans underwent oral treatment with indomethacin, a non‐steroidal anti‐inflammatory drug (NSAID) causing gastrointestinal injury.^[^ ^95^ ^]^

Indomethacin increased the intestinal permeability in healthy humans evaluated by using lactulose/rhamnose urine test.^[^ ^96^ ^]^ Ingestion of colostrum as supplement in diet attenuated the indomethacin‐induced intestinal permeability increase only in healthy volunteers but not in indomethacin‐prescribed patients.^[^ ^96^ ^]^ Moreover, oral administration of recombinant human Lf ameliorated the indomethacin‐induced intestinal permeability increase just in the small intestine not in gastroduodenal mucosa as documented in healthy human volunteers.^[^ ^97^ ^]^

It is known intestinal permeability injury triggers endotoxin translocation from the intestinal lumen to blood circulation causing systemic inflammation as documented in experimental trial in human volunteers.^[^ ^98^ ^]^ Therefore, clinical protocols have evidenced the effect of colostrum on attenuating the endotoxin‐induced intestinal permeability increase measured by the plasma concentration of endotoxin and zonulin as markers of permeability in critically ill patients.^[^ ^99^ ^]^

Under physical and emotional conditions found during exercise is triggered the response of stress hormones like glucocorticoids that have a critical role on the intestinal permeability and oral colostrum may provide benefits on intestinal wealth.^[^ ^100^ ^]^ Outcome of colostrum on ameliorating the increased intestinal permeability (measured by using lactulose‐rhamnose urinary test) has been reported in athletes underwent a cross‐over exercise protocol.^[^ ^101^ ^]^ Furthermore, colostrum reduced the intestinal permeability measured with lactulose‐rhamnose urine and stool zonulin tests in athletes during training.^[^ ^102^, ^103^ ^]^ Conversely, colostrum seems to increase, or has not effects all, on the intestinal permeability (measured by lactulose/rhamnose urine test or lactulose/mannose urine probes) as documented in healthy human volunteers underwent exercise protocol.^[^ ^104^, ^105^ ^]^

These controversial findings upon the impact of colostrum on intestinal permeability may be related with the timing of the colostrum sample tested; it seems that bovine colostrum composition over the first 3 days of lactation entail the decrease of biological activity as tested with lactulose/rhamnose ratio.^[^ ^106^ ^]^ Moreover, additional factors like divalent cations may influence the modulatory effect of colostrum on gut barrier. In this regard, in healthy human volunteers underwent heavy exercise program, oral ingestion of zinc along with colostrum mitigated the increased intestinal permeability measured by lactulose/rhamnose and HRP assays and strengthened the gut barrier evidenced by the TJPs increase.^[^ ^107^ ^]^

According to all above several factors like timing, cationic divalent content, and so on should be taking in mind to maximize the beneficial properties of colostrum on gut barrier tested for humans.

## 6 Concluding Remarks

This review may provide foundations on the design of pharmaceutical products containing colostrum ingredients such as bLf to enhance the therapeutic efficiency and/or reduce the dosage of drugs prescribed for the treatment of disease of clinical relevance including ulcerative colitis. At present, the properties of bovine milk protein derivatives on intestinal permeability have been scarcely documented in humans, but evidence raised from clinical trials provide promising findings of potential application of colostrum to control of the intestinal permeability in critically ill patients, users of non‐steroid anti‐inflammatory drugs, like athletes and militia members.

## Conflict of Interest

The authors declare no conflict of interest.

## Author Contributions

F.G.‐M. contributed in the literature search, design and preparation of figures, edited and revised the original drafts, and the final version of the manuscript; D.E.M.‐T., X.V.‐H., E.S.‐M. participated in the preparation of original draft; M.G.‐V. contributed in Table preparation and in original draft; R.O.‐P., participated in the preparation of the original draft; M.E.D.‐S. contributed in the paper conceptualization, literature curation, drafting of original paper.
